# Intraspecific chemodiversity provides plant individual- and neighbourhood-mediated associational resistance towards aphids

**DOI:** 10.3389/fpls.2023.1145918

**Published:** 2023-04-04

**Authors:** Dominik Ziaja, Caroline Müller

**Affiliations:** Chemical Ecology, Bielefeld University, Bielefeld, Germany

**Keywords:** associational resistance, aphid attraction, aphid abundance, common garden experiment, common tansy, plant-herbivore interactions, terpenoids

## Abstract

Some plant species express an extraordinarily high intraspecific diversity in phytochemicals (= chemodiversity). As discussed for biodiversity, higher chemodiversity may provide better protection against environmental stress, including herbivory. However, little is known about whether the resistance of a plant individual towards herbivores is mostly governed by its own chemodiversity or by associational resistance provided by conspecific neighbours. To investigate the role of chemodiversity in plant-aphid interactions, we used the Asteraceae *Tanacetum vulgare*, whose individuals differ pronouncedly in the composition of leaf terpenoids, forming distinct chemotypes. Plants were set up in a field consisting of plots containing five individuals of either the same or different chemotypes. Presence of winged aphids, indicating attraction, and abundance of winged and unwinged aphids, indicating fitness, were counted weekly on each plant. During the peak abundance of aphids, leaf samples were taken from all plants for re-analyses of the terpenoid composition and quantification of terpenoid chemodiversity, calculated on an individual plant (Shannon index, Hs_ind_, also considered as α-chemodiversity) and plot level (Hs_plot_, = β-chemodiversity). Aphid attraction was neither influenced by chemotype nor plot-type. The real-time odour environment may be very complex in this setting, impeding clear preferences. In contrast, the abundance was affected by both chemotype and plot-type. On average, more *Uroleucon tanaceti* aphids were found on plants of two of the chemotypes growing in homogenous compared to heterogenous plots, supporting the associational resistance hypothesis. For *Macrosiphoniella tanacetaria* aphids, the probability of presence differed between plot-types on one chemotype. Terpenoid chemodiversity expressed as a gradient revealed negative Hs_plot_ effects on *U. tanaceti*, but a positive correlation of Hs_ind_ with *M. tanacetaria* abundance. Aphids of *M. fuscoviride* were not affected by any level of chemodiversity. In conclusion, this study shows that not only the chemotype and chemodiversity of individual plants but also that of conspecific neighbours can influence certain plant-herbivore interactions. These effects are highly specific with regard to the plant chemotype and differ between aphid species and their morphs (winged vs. unwinged). Furthermore, our results highlight the importance of analysing chemodiversity at different levels.

## Introduction

Plants show a huge variation in natural products both interspecifically and intraspecifically, which affects interactions with other organisms across different trophic levels (Moore et al., 2014; Glassmire et al., 2016; Schneider et al., 2021). Such phytochemical diversity, also called chemodiversity (Müller and Junker, 2022), is recently increasingly studied, combining omics approaches with ecological concepts and applying diversity indices used in biodiversity research (Hilker, 2014; Wetzel and Whitehead, 2020). Frameworks for chemodiversity research have been developed that highlight the functional levels of plant chemodiversity and the spatial and temporal factors influencing it (Kessler and Kalske, 2018). Plants are confronted with numerous generalist and specialist antagonists and are thus subjected to a multitude of selective pressures. Across-species comparisons within plant clades revealed that generalist herbivores may promote the synthesis of repellent rather than attractive compounds (Salazar et al., 2018). With more specialised plant-herbivore interactions and at local scales with tight associations, the chemical dissimilarity within the plant community tends to increase (Becerra, 2007). Generally, the associational resistance hypothesis postulates that plants in communities with high biodiversity experience lower herbivore infestations than plants in less diverse communities (Tahvanainen and Root, 1972; Randlkofer et al., 2010). The chemodiversity among and within plant species may be a main driver for these effects. Indeed, increasing chemodiversity was found to reduce herbivore damage (Richards et al., 2015; Bustos-Segura et al., 2017), but the outcome also depends on the specific environmental conditions and characteristics of the local herbivore communities (Fernandez-Conradi et al., 2021). In certain plant communities, chemodiversity of low-volatile compounds reduced herbivory by generalists, while specialist herbivory was reduced by high-volatile compounds (Salazar et al., 2016), such as monoterpenoids. So far, research has mostly focused on either community-wide interspecific chemodiversity (sometimes also called γ-chemodiversity, Kessler and Kalske, 2018) or individual-based intraspecific chemodiversity (considered as α-chemodiversity), but little is known on the effects of community-wide intraspecific chemodiversity (β-chemodiversity) on plant-herbivore interactions (but see, e.g., Bustos-Segura et al., 2017).

Terpenoids are the most diverse group of plant natural products, consist of isoprene (C_5_H_8_) precursors and are often stored in glandular trichomes on the leaf surface of, for example, Asteraceae (Gershenzon and Dudareva, 2007; Aschenbrenner et al., 2013). Terpenoids are either stored and emitted constitutively or induced upon exposure to abiotic or biotic stresses (Gershenzon and Dudareva, 2007). Against herbivores, they can act directly as repellent or deterrent or impact the development and reproduction. However, these compounds can also attract specialists or be involved in indirect plant defence by attracting natural enemies of herbivores (Gershenzon and Dudareva, 2007; Boncan et al., 2020). Plant fitness-enhancing effects of terpenoids may also occur on a community level. Semi-volatile sesquiterpenoids of different hydrophobicity emitted from one plant were found to be adsorbed and re-emitted by its allospecific neighbours, repelling herbivores and thus providing associational resistance, while for a highly volatile monoterpene this effect could not be observed (Himanen et al., 2010). Such mixed blends across neighbouring plants may disturb the host localisation of insects, because they likely use the specific ratio of compounds in volatile blends for this purpose (Bruce et al., 2005). The real-time odour environment of an individual may in the end determine whether it is attracted to its host or distracted by repellent effects (Shao et al., 2021).

In aphids, host localisation is mainly performed by the winged morphs. Compared to unwinged morphs, winged morphs of *Rhopalosiphum padi* were shown to have a higher expression of genes involved in olfactory systems and thus a higher sensitivity towards volatile compounds (Peng et al., 2020), playing a critical role in host colonisation. A peak of winged aphid morphs can usually be observed in early summer when colonisation takes place in a more dispersed pattern. Subsequently, unwinged aphids become more dominant and colonisation occurs more locally (Dixon, 1977; Braendle et al., 2006; Mehrparvar et al., 2013). Despite winged morphs being potentially more sensitive to volatile plant metabolites, both morphs can be affected by metabolites located on the leaf surface, in the leaf and in the phloem sap (Wink et al., 1982; Powell et al., 2006; Chang et al., 2022). Terpenoids have been shown to attract or repel aphid species (Webster et al., 2008; Kos et al., 2013), while terpenoids stored in glandular trichomes negatively affected fecundity and longevity of aphids (Wang et al., 2021). Apart from the terpenoids, the nutritional value of the phloem sap is particularly important for aphids and determines whether aphids will stay on the plant and be able to reproduce (Karley et al., 2002; Nowak and Komor, 2010).

The perennial Asteraceae common tansy, *Tanacetum vulgare* L., is an aromatic plant species that shows a pronounced intraspecific chemodiversity of mono- and sesquiterpenoids (Keskitalo et al., 2001; Judzentiene and Mockute, 2005; Wolf et al., 2011), which are passed on genetically (Holopainen et al., 1986; Keskitalo et al., 2001). Based on their relative composition, terpenoids are used to assign tansy individuals to specific mono-chemotypes with one predominant terpenoid or mixed-chemotypes with one to two additional major satellite terpenoids (Holopainen et al., 1986). In nature, different chemotypes often occur together in patches next to each other (Kleine and Müller, 2011). Tansy is visited by numerous herbivores (Schmitz, 1998), including various aphid species that are mono- or oligophagous on tansy. The chemotypes have been revealed to play a crucial role in interactions between tansy and its specialised herbivores (Kleine and Müller, 2011; Jakobs and Müller, 2018; Benedek et al., 2019). For example, winged morphs of *Metopeurum fuscoviride* preferably colonise chemotypes emitting α-thujone, (*E*)-dihydrocarbone, α-copaene and β-cubebene (Clancy et al., 2016), whereas unwinged morphs of *Uroleucon tanaceti* use most likely terpenoids to locate preferred plant parts within a plant individual (Jakobs and Müller, 2019). Moreover, tansy chemotypes also affect aphid populations directly by impacting their growth rate (bottom-up) and indirectly by influencing the establishment of different arthropod food webs (top-down), resulting in chemotype-specific communities of aphids, their predators and mutualistic ants (Balint et al., 2016; Senft et al., 2018). In turn, aphids can also modulate the nutritional quality of the phloem sap with changes depending on the aphid species, the plant part as well as the chemotype (Jakobs et al., 2019). Thus, this plant species offers a highly suitable model to study the role of chemodiversity in plant-aphid interactions.

This study investigated how certain leaf chemotypes and varying degrees of intraspecific chemodiversity on both individual plant and group level (= direct neighbourhood) of tansy plants affect the attraction and occurrence (presence and abundance on a host plant) of aphids specialised on this plant species. A common garden experiment was set up containing homogenous and heterogenous groups (= plots) of five plant individuals per plot belonging to one or five chemotypes and aphid presence per plant was scored across the season. Chemodiversity was considered on the plant individual level (= α-chemodiversity) and on the plot level (= β-chemodiversity). We hypothesised that certain chemotypes are more attractive to winged aphids than other chemotypes and that homogenous chemotype plots are more attractive than heterogenous plots, potentially due to a less mixed odour bouquet facilitating host localisation in the former and associational resistance in the latter. These effects may differ depending on the aphid species. Finally, we expected that plants and plots with a lower chemodiversity enhance the occurrence of aphids on plant individuals, used as a proxy for aphid fitness.

## Materials and methods

### Experimental set-up

A stock of tansy plants was established from seeds collected in January 2019 at four different sites in Bielefeld, Germany (51°58’58.52’N, 8°27’12.27’E; 51°58’51.8’N, 8°27’40.0’E; 51°58’59.3’N, 8°28’13.8’E; 51°58’42.2’N 8°28’35.5’E; elevation 98-105 m). Seeds were collected from 8-10 mother plants per site with at least 20 m distance between each plant, to enhance the likelihood to collect seeds from genetically distinct individuals (tansy can grow clonally). Seeds were germinated and terpenoid profiles determined from young leaves as described in 2.3. Based on the monoterpenoid profiles, plants of five distinct chemotypes were picked to build up the stock collection. The terpenoid composition of two of these chemotypes was dominated by a single monoterpenoid (> 55% of total terpenoid concentration, mono-chemotypes), either artemisia ketone (called “Keto” chemotype in the following) or β-thujone (“BThu”). The composition of the other three chemotypes was dominated by two to three compounds (10 – 50% of total terpenoid concentration, mixed-chemotypes), either α-thujone and β-thujone (“ABThu”), artemisyl acetate, artemisia ketone and artemisia alcohol (“Aacet”) or (*Z*)-myroxide, santolina triene and artemisyl acetate (“Myrox”) (in total *n* = 30 plants per chemotype, originating from different mother plants, called chemo-genotypes hereafter). Two clones from each of these plants were produced from rhizome cuttings, grown in a greenhouse and transferred outside into a mixed soil:sand-bed from September to December 2019 for acclimatisation, and kept there until the last week of May 2020. Then, these non-flowering plants were introduced into a field common garden.

The common garden was set up close to Bielefeld University (52°03’39.43’N, 8°49’46.66’E; elevation 142 m). Of each of in total 150 chemo-genotypes, one clone was planted in a homogenous plot consisting of five plants of the identical chemotype (*n* = 30 plots; 6 plots per chemotype). The other clone was planted in a heterogenous plot (*n* = 30 plots) consisting of one plant of each of the five chemotypes. The site (24 x 17 m) was split into six blocks, each consisting of ten plots (1 x 1 m) with 1 m between plots and 2 m between blocks (for detailed set-up see **Supplementary Figure S1**). Plants were planted in PVC-tubes (diameter 16 cm, height 30 cm), which were inserted 25 cm deep into the soil to allow for distinction of individual plants (referred to as pots from here on). Within plots, pots were arranged in a circle with equidistance between neighbouring plants. All plants within a plot were descendants from different maternal plants.

The area between blocks and plots was milled once in spring and once in autumn every year. Throughout the season, vegetation within plots was removed manually if it reached half the length of the maximum tansy plant height in the respective plot. When occurring, seedlings of additional tansy plants were removed. In December 2020, the aboveground biomass of all plants was harvested down to 5 cm above ground, thereby homogenising plant growth once and potentially removing overwintering aphid eggs, thus enhancing the chance that plants were colonised by migrating aphids in 2021.

### Scoring of aphids

Scoring of aphids visiting the experimental tansy plants took place on a weekly basis from May 6^th^ 2021 until August 18^th^ 2021, when almost no aphids were observed anymore. It was performed usually mid-week within two days between 8 am and 6 pm. For scoring of aphid species specialised on tansy, each plant was carefully examined, trying to prevent the aphids from dropping off the plant. Winged and unwinged individuals per species were counted separately. The presence (yes/no) of winged aphids on a plant or plot was considered as “attraction”. Observing at least one aphid of any morph on a plant individual was considered as species presence (yes/no). Aphid counts (= abundance) were taken as an indication of aphid fitness. Because *U. tanaceti* develops huge colonies, extra measures were implemented: (a) counting was capped when a colony on a plant reached 2000 individuals; (b) starting on June 30^th^ aphid populations with a count ≥ 100 were estimated in increments of ten. To account for the variance explained by the presence of ants for ant-tended aphid species (*M. fuscoviride*), the presence of ants in plant pots and ants actively harvesting honeydew from aphids were noted down. Furthermore, ant nests in the ground either within or directly around the plant pot were scored after manually checking for ant activity by poking into the nest using a stick. Every week, the sampling order and person counting (*n* = 5) were randomised on the plot-level.

### Plant phenotyping

On June 21^st^-22^nd^ 2021, all plants were phenotyped, assessing the length of the highest shoot, the number of shoots, the number of leaves and the number of stems with inflorescences per plant. For later analyses of the actual terpenoid composition, the top 4 cm of the youngest fully developed, non-infested leaf of one of the stems was cut off and immediately frozen in liquid nitrogen. Leaf samples were harvested between 10 am and 1:30 pm. Most except ten plants had not developed flowerheads at that time point. One plant of a homogenous Myrox plot turned out to belong to a different chemotype than originally suggested. Thus, all data for this entire plot was excluded from all statistical analyses.

### GC-MS analysis of leaf terpenoid composition

The harvested leaf material was freeze-dried, homogenised, weighed and extracted in heptane. Samples were put in an ultrasonic bath for 5 min and then centrifuged. The supernatants were analysed using gas chromatography coupled with mass spectrometry (GC-MS; GC 2010plus – MS QP2020, Shimadzu, Kyoto, Japan) on a semi-polar column (VF-5 MS, 30 m length, 0.2 mm ID, 10 m guard column, Varian, Lake Forest, United States) in electron impact ionisation mode at 70 eV and with helium as carrier gas. Samples were injected at 240°C with a 1:10 split. A starting temperature of 50°C was kept for 5 min, ramping up to 250°C at 10°C min^-1^, then increasing with 30°C min^-1^ to a final temperature of 280°C, hold for 3 min. Blanks of heptane with the internal standard and an alkane standard mix (C7–C40, Sigma Aldrich, Taufkirchen, Germany) were measured regularly between sample batches. Terpenoids were identified based on their retention indices (RI) (van den Dool and Kratz, 1963) and by comparing spectra to synthetic reference compounds, where available, and to entries of the libraries NIST (National Institute of Standards and Technology, Gathersburg, USA, 2014), Pherobase (El-Sayed, 2012) and those reported in reported in Adams (2007). Terpenoids were quantified based on the peak area of the total ion chromatogram and the relative composition determined by dividing each peak area by the sum of the peak areas of all peaks within each sample.

### Statistical analyses

All statistical analyses were carried out in R version 4.2.1 (R Core Team, 2022), using the packages vegan (Oksanen et al., 2022), dplyr (Wickham et al., 2021), glmmTMB (Brooks et al., 2017), DHARMa (Hartig, 2021), car (Fox and Weisberg, 2019), insight (Lüdecke et al., 2019), emmeans (Lenth, 2021), pgirmess (Giraudoux, 2022) and glmnet (Friedman et al., 2010). Visualisations were made in ggplot2 (Wickham, 2016). R-scripts used for statistical analysis and a virtual environment of the RStudio project are available on github (https://github.com/DoZi93/CommonGarden-aphid-2021).

Aphid species were analysed separately to keep the complexity manageable, avoid overfitting of models and to specify models according to the ecology of individual aphid species. The occurrence of winged aphids (attraction) per plant and total count (abundance) per week were used for further analyses. To exclude zero-values simply due to the absence of an aphid species, all data recorded for every species (winged presence, total count) was filtered across weeks based on the cumulative sum over the season; the first week exhibiting ≥ 1% was selected as first, the week displaying the elbow of the curve towards the end of the season was selected as last week. If nymphs of *U. tanaceti* with signs of wing-forming were observed on a plant during a counting event, their count was included in the total aphid count but set to zero for all analysis targeting winged aphid presence, because they were likely produced by aphids that had already colonised the plant. This approach was not applied to *M. fuscoviride* and *M. tanacetaria*, since no winged nymphs of these species were found. For all generalised linear mixed models (GLMM) of total counts, goodness of fit and the appropriate distribution (Poisson, negative binomial 1, negative binomial 2) were evaluated based on simulated residuals using DHARMa plots. Occurrence of winged aphids was binary data and therefore analysed using binomial distribution. Random effects causing convergence problems due to low variance explained were dropped from the models.

Non-transformed whole season total count data of every species was analysed using zero-inflated generalised linear mixed models (zi-GLMM) after checking for zero-inflation. The aphid presence (at least one winged or unwinged individual observed on plant) was modelled by the zero-inflation component of the total count models. In all models, chemotype, plot-type, the chemotype x plot-type interaction and calendar week were implemented as fixed effects. Since *M. fuscoviride* is ant-tended, presence of ants, ant nests and ants actively tending aphids were included as binary, fixed effects for the models calculated for this aphid species. Block, plot number, plant clone ID, plant ID, maternal genotype and observer were included as random effects in every model. Nestedness of random effects was accounted for by uniquely coding the nested random effects (block and plot number). The zero-inflation model component was modelled with the same fixed and random effects as the conditional model.

For the week in which plant morphological and chemical data were sampled (June 2021), the relative abundance of each terpenoid was compared within chemotype between plants grown in homogenous versus heterogenous plots using pairwise Mann-Whitney U tests. The resulting *p*-values were adjusted using the Holm-method. In addition, effects of plant traits on winged aphid presence and the total count of aphids were analysed on the individual plant-and plot-level using non-zero inflated GLMMs. The individual plant-level chemodiversity Hs_ind_ was quantified by calculating the Shannon diversity index $H_{s}=-\sum^{}p_{i}\times ln\;p_{i}$ (Shannon and Weaver, 1964), with *p* being the relative abundance of each terpenoid within an individual (sampled in June 2021). For an individual plant-level analysis, ln (x+1)-transformed morphological parameters, namely length of highest shoot, number of stems, number of leaves and number of stems with inflorescences, as well as Hs_ind_ were included in the models as fixed effects and the same random effects, except for plant ID, were used as in the whole season GLMMs. To assess effects on the plot-level, data was summarised for each plot: presence of winged aphids was evaluated plot-wise. Total aphid count, number of leaves, number of shoots and number of shoots with inflorescences were summed up per plot; for the length of the highest shoots the average across plants per plot was taken. The plot chemodiversity Hs_plot_ was calculated with *p* being the average of the relative abundance of each terpenoid across the five plants per plot. Except for the aphid-related response variables and the Hs_plot_, variables calculated on plot-level were also ln (x+1)-transformed. The fixed effects were identical to those used in the individual plant-level models, while only block was included as random effect.

To infer the effects of individual terpenoids on aphid-tansy interactions, a Poisson-LASSO regression was applied to those total aphid counts, which were significantly affected by the chemodiversity (*Macrosiphoniella tanacetaria* by Hs_ind_, *U. tanaceti* by Hs_plot_), using the relative composition of each terpenoid from the individual plant (individual plant-level) or the average of each plot (plot-level) as predictor variables (Salazar et al., 2018). The penalisation term integrated in a LASSO regression allows for coefficients to be estimated zero, resulting in both feature selection and assessment of correlations between features and response variables (James et al., 2013). The total count of aphid species was analysed using a Poisson LASSO regression. Minimal lambda of all LASSO models was determined using K-fold cross validation.

## Results

### Effects of chemotype and plot-type on aphid attraction and occurrence

**Figure 1:**
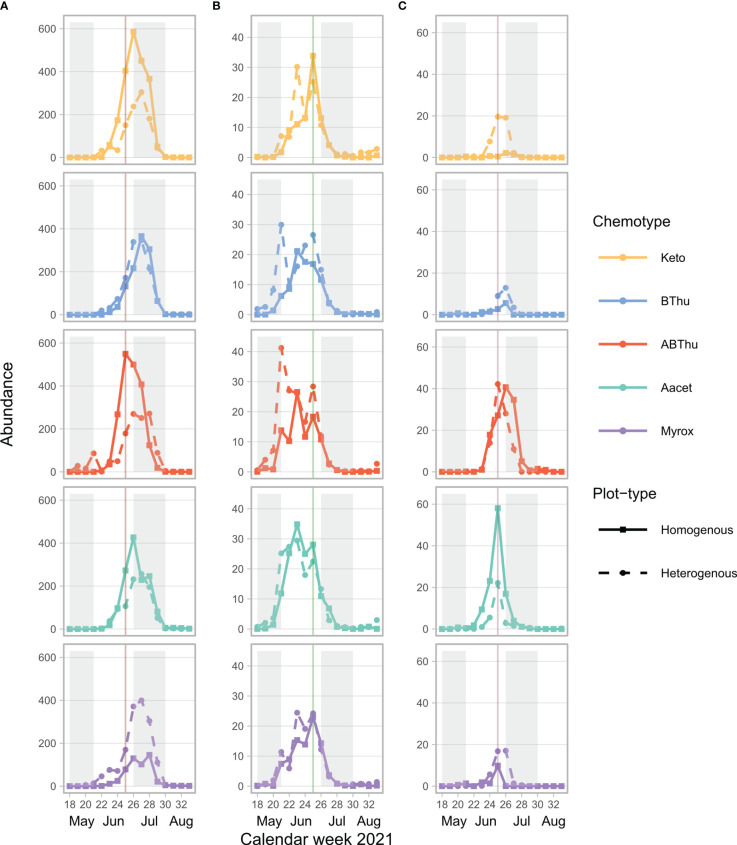
Mean total count of winged and unwinged aphids of **(A)** *Uroleucon tanaceti*, **(B)** *Macrosiphoniella tanacetaria* and **(C)** *Metopeurom fuscoviride* per calendar week on plants of *Tanacetum vulgare* grown in plots of five individuals of the same (homogenous) or different (heterogenous) chemotypes (Keto, artemisia ketone chemotype; BThu, β-thujone chemotype; ABThu, α-/β-thujone chemotype; Aacet, artemisyl acetate/artemisia ketone/artemisia alcohol chemotype; Myrox, (*Z*)-myroxide/santolina triene/artemisyl acetate chemotype) across the season. Vertical lines indicate the calendar week in which the morphological and leaf terpenoid characterisation of the plants took place; *n* = 25-30 per chemotype x plot-type combination.

**Figure 2:**
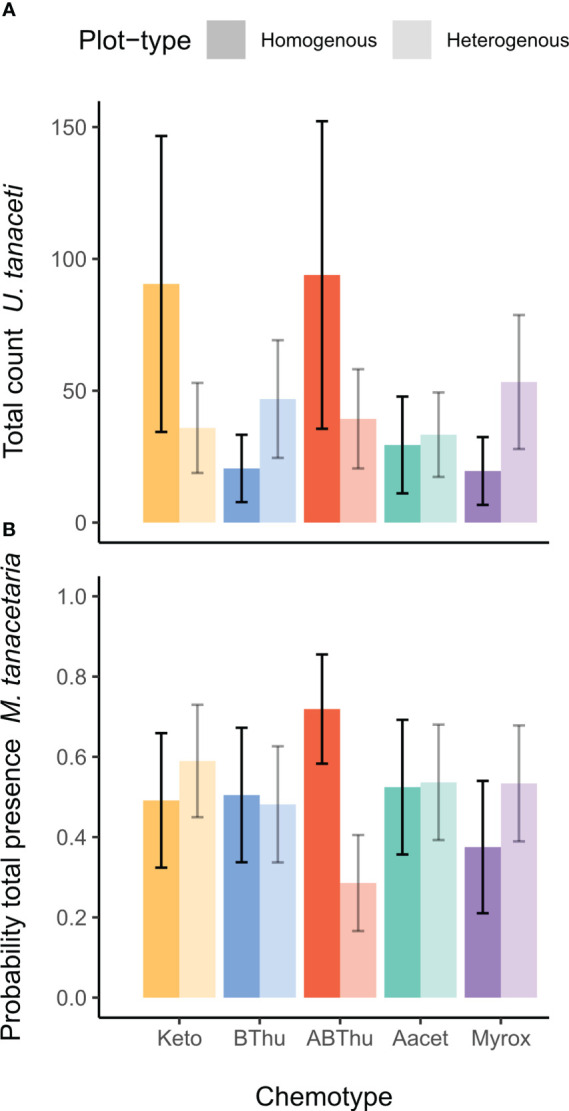
Em mean predictions of **(A)** the total count of winged and unwinged individuals of *Uroleucon tanaceti* and **(B)** the probability of presence of winged and unwinged individuals of *Macrosiphoniella tanacetaria* on plants of *Tanacetum vulgare* grown in plots of five individuals of the same (homogenous) or different (heterogenous) chemotypes (Keto, artemisia ketone chemotype; BThu, β-thujone chemotype; ABThu, α-/β-thujone chemotype; Aacet, artemisyl acetate/artemisia ketone/artemisia alcohol chemotype; Myrox, (*Z*)-myroxide/santolina triene/artemisyl acetate chemotype). A significant [**(A)**: *p* = 0.01, **(B)**: *p* = 0.02] chemotype x plot-type interaction was found based on the conditional **(A)** and zero-inflation **(B)** component of the zi-GLMM. Number of observations per chemotype x plot-type interaction *n*_obs_ = 200-240 and plants per chemotype x plot-type interaction *n*_plants_ = 25-30.

Across the season, the tansy specialist aphid species *U. tanaceti*, *M. tanacetaria* and *M. fuscoviride* were frequently found on the experimental plants. The attraction of winged aphids and the presence and total count of winged and unwinged morphs of these aphid species depended on the calendar week (*Χ^2^* ranging from 9.03 to 57049, *p* < 0.001) with the exceptions of winged morph presence and total presence of *M. fuscoviride* (**Table 1**). On average, higher numbers of *U. tanaceti* were observed on homogenous plots of the Keto and ABThu chemotype, whereas on the Myrox chemotype numbers were higher when this chemotype grew in heterogenous plots (**Figures 1A**, **2A** and **Table 1**). For *M. tanacetaria*, only the presence of winged and unwinged morphs was affected by the chemotype x plot-type interaction (**Figure 1B** and **Table 1**), i.e. there was a higher probability to observe the aphid species on ABThu plants in homogenous compared to heterogenous plots (**Figure 2B**). The total count of *M. fuscoviride* was positively affected by the presence of ants tending their colonies (model predictions: 3.70 ± 1.21 without ant-tending, 18.94 ± 6.14 with ant-tending), but not by plot-type or chemotype (**Figure 1C**, **Table 1**). Furthermore, across all whole season models the mother plants explained less than 1% of the variance of the datasets (**Table 1**). During their main abundance phase, *U. tanaceti* and *M. tanacetaria* were present on almost all plants of the different chemotype x plot-type interactions, whereas *M. fuscoviride* occupied at most 5-12 plants depending on the chemotype x plot-type combination (**Supplementary Figure S2**).

**Table 1:** (zi-)GLMM estimates of total count (unwinged and winged aphids) and presence of winged morphs of three aphid species specialised on *Tanacetum vulgare* across the counting season.

Aphid species	Sample size	Response variable	Chemotype	Plot-type	Week	Chemotype x Plot-type	Ant presence	Ant nest presence	Ant MF presence^3^	Block	Plot	Clone ID	Mother plant	Observer	Plant ID
			*d.f.* = 4	*d.f.* = 1	*d.f.* = 1	*d.f.* = 4	*d.f.* = 1	*d.f.* = 1	*d.f*. = 1	*d.f. =* 1	*d.f.* = 1	*d.f.* = 1	*d.f.* = 1	*d.f.* = 1	*d.f*. = 1
*Uroleucon tanaceti*	N_obs_ = 2360	Winged presence GLMM (bi)	4.62	0.77	27.60***	2.76	—	—	—	*1.00*	*0.84*	*1.14*	*<0.0001*	*1.00*	*<0.0001*
	N_obs_ = 2360	Total count	5.16	0.06	58660.02***	13.50**	—^1^	—	—	*3.56*	*24.26*	*4.95*	*0.51*	*13.18*	*10.36*
		zi-GLMM (p)	6.42	0.55	462.41***	4.73	—	—	—						
*Macrosiphoniella tanacetaria*	N_obs_ = 1770	Winged presence GLMM (bi)	1.06	<0.0001	12.94***	3.99	—	—	—	*0.36*	*<0.0001*	*<0.0001*	*0.29*	*0.72*	*<0.0001*
	N_obs_ = 2360	Total count	6.91	<0.0001	155.71***	2.03	—	—	—	*29.54*	*17.65*	*5.02*	*x^2^*	*17.08*	*36.94*
		zi-GLMM (p)	11.25*	0.01	402.04***	12.16*	—	—	—						
*Metopeurum fuscoviride*	N_obs_ = 2065	Winged presence GLMM (bi)	6.38	1.50	0.58	2.09	7.29**	1.19	66.70***	*4.95*	*8.46*	*0.99*	*<0.0001*	*3.65*	*0.77*
	N_obs_ = 1770	Total count	3.80	1.46	10.73**	8.43	0.81	0.01	41.60***	*x*	*7.65*	*5.82*	*x*	*x*	*12.23*
		zi-GLMM (nb2)	3.17	0.36	0.01	1.38	17.12***	3.03	<0.0001						

po: response variable was modelled using a Poisson distribution.

bi: response variable was modelled using a binomial distribution.

nb2: response variable was modelled using a negative binomial 2 distribution.

^1^Effects marked with long hyphen were left out of the model due to being inapplicable for the model.

^2^Random effects marked with “x” were dropped due to causing model convergence issues because of the low variance explained.

^3^“Ant MF presence” indicates if at the time point of counting ants were observed on a *Metopeurum fuscoviride* colony.

Shown are the Χ^2^ estimates based on Wald’s type 3 chi-square test (regular font) and the proportion of variance explained by random effects (italic). Numbers in bold font indicate significant effects with asterisks displaying the significance level (*p < 0.05; **p < 0.01; ***p < 0.001). For zero-inflation model estimates of the conditional model are displayed in the first line, estimates of the zero-inflation model in the second line.

### Terpenoid composition, differences between plot-types and chemodiversity Hs_ind_ and Hs_plot_

**Figure 3:**
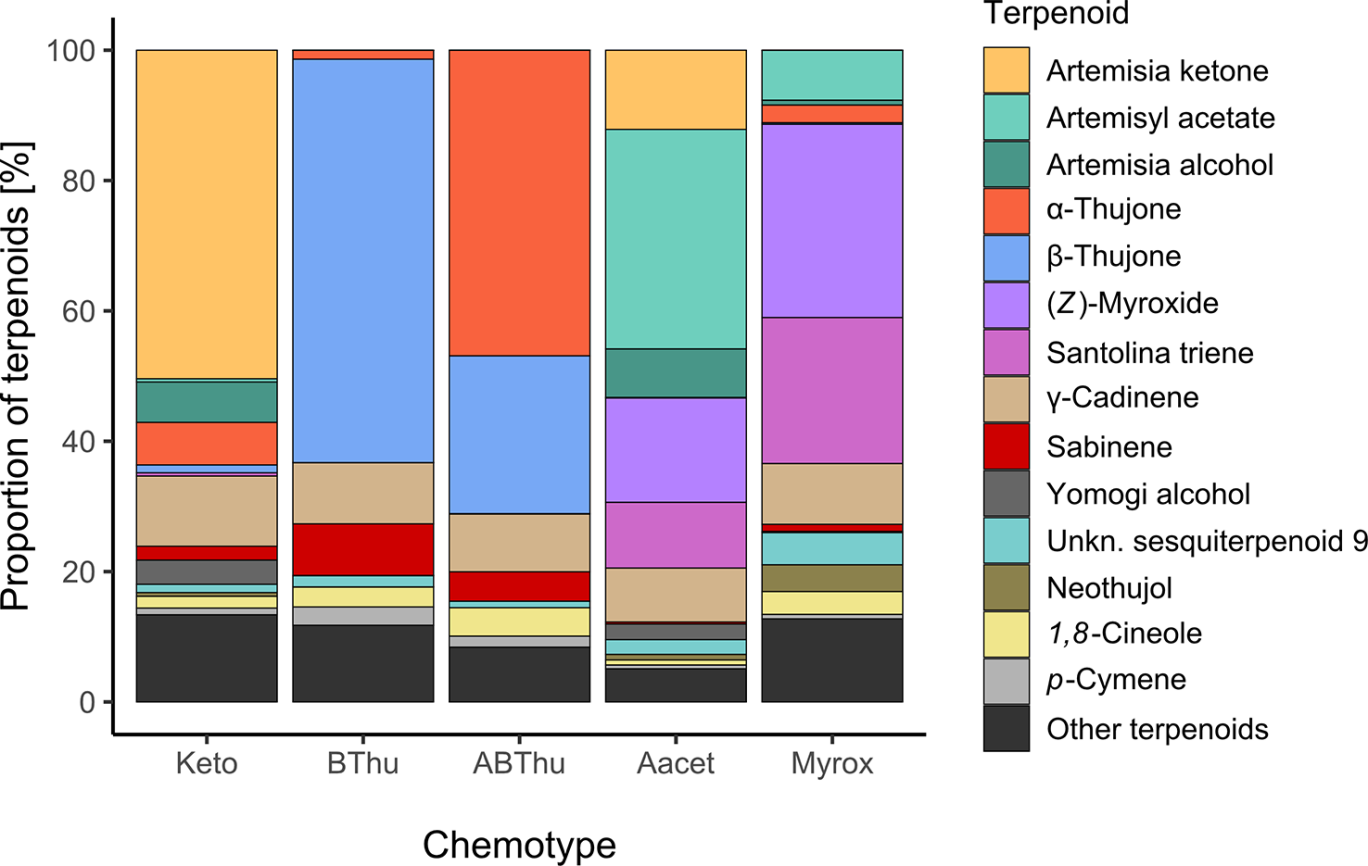
Mean terpenoid composition of leaves of *Tanacetum vulgare* of five different chemotypes grown in the common garden, sampled in June 2021. All terpenoids are individually displayed that account together for ≥ 80% of the total chemotype composition; *n* = 55-60 individuals per chemotype.

In total, 52 terpenoids were found in leaves throughout all plants (**Supplementary Table S1**). The monoterpenoid(s) originally dominating each chemotype at initial terpenoid analyses (measured at seedling stage) continued to represent > 50% of the terpenoid composition per chemotype when analysed from the plants grown in the field (**Figure 3**). Some monoterpenoids dominating in one chemotype could also be found across multiple chemotypes. For example, the Keto chemotype showed on average small proportions of *α*-thujone (6.5%), while the Aacet chemotype contained santolina triene (10.1%) as well as (*Z*)-myroxide (16.0%). Other monoterpenoids present in higher proportions were sabinene (BThu 7.9%, ABThu 4.5%) and 1,8-cineole (ABThu 4.4%, Santo 3.5%, BThu 3.1%). Regarding sesquiterpenoids, in every chemotype γ-cadinene (8.3 – 10.8%) and an unknown sesquiterpenoid (RI 1673, 1.0 - 4.9%) were present.

Across all chemotypes, significant differences in the proportion of individual terpenoids were found between plants growing in homo- vs. heterogenous plots (**Supplementary Figures S3–S7**). For example, plants of the Keto chemotype grown in homogenous plots showed on average a significantly higher proportion of (*E*)-sabinene hydrate and a sesquiterpenoid compared to plants grown in heterogenous plots (**Supplementary Figure S3**). The relative composition differed in plants of the Aacet chemotype in more than a dozen terpenoids between plants of different plot-types, most notably significantly higher proportions of artemisia ketone, santolina triene, 1,8-cineol, sabinene and neothujol and significantly lower proportions of γ-cadinene in plants of homogenous plots (**Supplementary Figure S6**). Plants of the Myrox chemotype grown in homogenous plots had a significantly higher proportion of artemisia alcohol and a significantly lower proportion of γ-cadinene (**Supplementary Figure S7**).

In homogenous and heterogenous plots the average Hs_ind_ ranged between 1.16 and 2.01, with the lowest value found in plants of the BThu chemotype grown in heterogenous plots. On the plot level, the average Hs_plot_ was with 2.51 highest in plants grown in heterogenous plots (**Table 2**).

**Table 2:** Shannon-diversity (Hs) of the five tested chemotypes calculated based on the relative terpenoid composition obtained from GC-MS measurements of youngest, fully developed leaf samples taken in calendar week 25 from each plant planted in the common garden.

Level	Plot-type	Chemotype	Mean	Sd
Individual plant level (Hs_ind_)	Homogenous	Keto	1.52	0.48
		BThu	1.39	0.60
		ABThu	1.39	0.48
		Aacet	2.00	0.33
		Myrox	1.85	0.45
	Heterogenous	Keto	1.43	0.38
		BThu	1.16	0.51
		ABThu	1.49	0.38
		Aacet	1.58	0.34
		Myrox	2.01	0.39
Plot level (Hs_plot_)	Homogenous	Keto	1.95	0.22
		BThu	1.67	0.35
		ABThu	1.61	0.32
		Aacet	2.21	0.22
		Myrox	2.24	0.27
	Heterogenous	—	2.51	0.17

Mean and standard deviation (Sd) are calculated across either each individual plant (Hs_ind_; *n* = 25-30 per chemotype x plot-type combination) or the average across five plants per plot (Hs_plot_; *n* = 4-5 per homogenous chemotype and heterogenous plots). For composition of chemotypes see **Figure 3**.

### Effects of individual plant- and plot-level morphology as well as Hs_ind_ and Hs_plot_ on aphid occurrence and attraction

In the week in which leaf sampling took place, winged aphids of *U. tanaceti*, *M. tanacetaria* and *M. fuscoviride* were observed on 162, 82 and 28 of the 291 plants from which terpenoids were re-analysed, respectively. On the individual plant-level, the likelihood of observing winged aphids (attraction) of *U. tanaceti* was reduced by the number of shoots (*X^2^* = 3.99; *p* = 0.046) but increased by the number of leaves (*X^2^* = 7.29; *p* = 0.01). Winged aphids of *M. tanacetaria* and *M. fuscoviride* were not affected by any morphological plant trait on both individual plant- and plot-level (**Supplementary Table S2**). Moreover, Hs_ind_ and Hs_plot_ did not show any effect on the presence of winged aphids (**Supplementary Table S2**).

**Figure 4:**
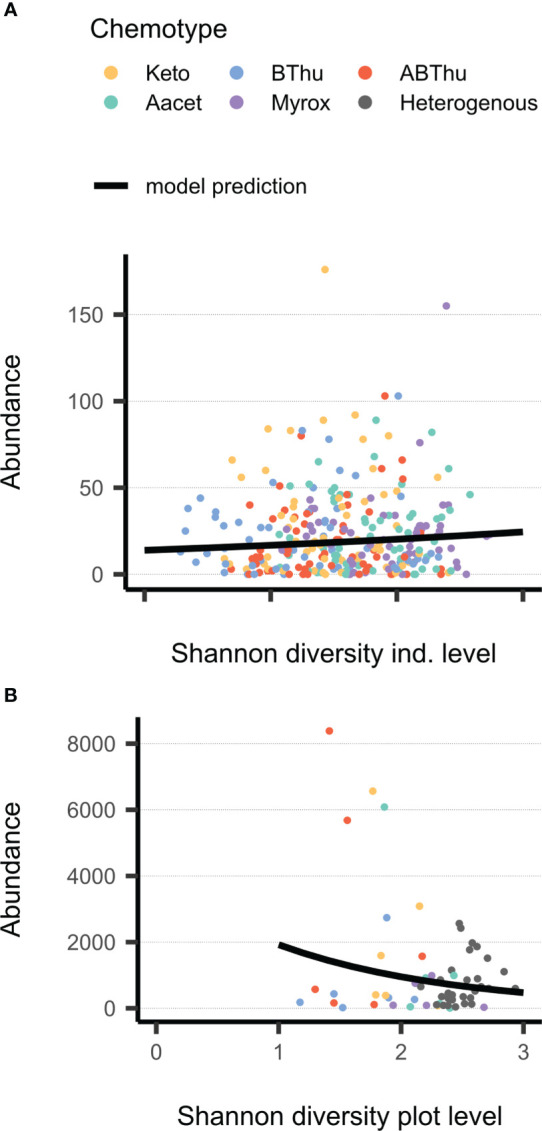
Relationship between Shannon diversity of terpenoids in leaves on **(A)** plant individual level (Hs_ind_) or **(B)** plot level (Hs_plot_) and total count of aphids (winged and unwinged) in June 2021 on *Tanacetum vulgare* plants of five different chemotypes grown in plots of five individuals of the same (homogenous) or different (heterogenous) chemotypes (Keto, artemisia ketone chemotype; BThu, β-thujone chemotype; ABThu, α-/β-thujone chemotype; Aacet, artemisyl acetate/artemisia ketone/artemisia alcohol chemotype; Myrox, (*Z*)-myroxide/santolina triene/artemisyl acetate chemotype). **(A)** Count of *Macrosiphoniella tanacetaria* on every individual plant; *n*_plants_ = 55-60 per chemotype. **(B)** Cumulative count of *Uroleucon tanaceti* for every plot; *n*_plots_ = 5-6 (per homogenous plots) or 30 (heterogenous plots).

The total count (abundance) of both *U. tanaceti* and *M. fuscoviride* was positively affected by the number of leaves on the individual plant-level. Regarding the plot-level, plant shoots were more influential than the leaves: *U. tanaceti* was positively affected by the shoot height, *M. fuscoviride* was negatively affected by the number of shoots, while numbers of leaves had no effect. Next to morphological traits, the total count was also affected by plant chemodiversity. On the individual level, Hs_ind_ affected the total count of *M. tanacetaria* positively (**Table 3**, **Figure 4A**). On the plot level, Hs_plot_ had a negative influence on the total aphid count of *U. tanaceti* (**Table 3**, **Figure 4B**). For both of these significant effects, the LASSO regression estimated the coefficients of all 52 individual terpenoids to be zero, i.e., no linear combination of terpenoids could predict these two counts.

**Table 3:** GLMM-estimates of total count (winged + unwinged morphs) of aphid species specialised on *Tanacetum vulgare* in calendar week 25 of 2021.

Response variable		*Uroleucon tanaceti*				*Macrosiphoniella tanacetaria*				*Metopeurum fuscoviride*			
		Total count (abundance)^b^				Total count (abundance)^a^				Total count (abundance)^a^			
Level		Individual plant level		Plot level		Individual plant level		Plot level		Individual plant level		Plot level	
Fixed Effects	*d.f.*	*Χ^2^*	Effect	*Χ^2^*	Effect	*Χ^2^*	Effect	*Χ^2^*	Effect	*Χ^2^*	Effect	*Χ^2^*	Effect
Shannon diversity	1	0.61	–	3.90*	↓^5^	3.90*	↑	0.15	–	1.00	–	0.25	–
Shoot length	1	0.87	–	8.28**	↑	0.02	–	3.17	–	2.08	–	0.25	–
Number of shoots	1	0.03	–	<0.0001	–	0.02	–	0.004	–	0.01	–	10.29**	↓
Number of leaves	1	16.24***	↑^4^	0.31	–	3.31	–	0.45	–	4.75*	↑	0.94	–
Number of shoots with inflorescences	1	—^2^	—	—	—	0.14	–	<0.0001	–	0.15	–	1.94	–
Ant presence	1	—	—	—	—	—	—	—	—	52.49***	↑	14.57***	↑
Random Effects		Variance [%]^3^		Variance [%]		Variance [%]		Variance [%]		Variance [%]		Variance [%]	
Block	1	*2.72*		*17.65*		*18.65*		*21.50*		*<0.0001*		*<0.0001*	
Plot	1	*29.65*		—		*13.77*		—		*21.24*		—	
Mother plant	1	*<0.0001*		—		*1.24*		—		*x^6^*		—	

a response variable was modelled using a negative binomial 1 distribution.

b response variable was modelled using a negative binomial 2 distribution.

^1^ *p*-values were estimated using a type-3 Wald chi-square test.

^2^Effects marked with long hyphen were left out of the model due to being inapplicable for the model.

^3^Variance [%] of random factors displays the percentage of the total variance that is explained by the random factors.

^4^↑ display a positive relationship with the fixed effect.

^5^↓ displays a negative relationship with the fixed effect.

^6^Random effects marked with “x” were left out of the model due to causing model convergence problems.

The *Χ^2^* estimates are based on Wald’s type 3 chi-square test, numbers in bold font indicate significant effects, asterisks display the significance level (*p< 0.05; **p< 0.01; *** *p*< 0.001). Results of winged presence were moved into the supplement since no significant effects were found. Total number of plants on which aphids were scored: 291.

## Discussion

We assessed the effects of intraspecific chemodiversity of plants, here in the form of differently composed leaf terpenoids, on the presence of winged aphids, indicating attraction, as well as total aphid count, indicating fitness. These effects were assessed on the individual plant as well as on the plant neighbourhood level. Overall, our results showed that chemodiversity mainly affected the presence or abundance of aphids on the plants, but not the attraction of winged aphids. Furthermore, for the affected aphid species, these effects were highly specific in terms of the plant chemotype and its respective neighbourhood.

The attraction of all three detected aphid species specialised on tansy was neither affected by the chemotype, plot-type, nor their interaction, in contrast to our expectations. First, we had hypothesised that aphids would find certain chemotypes more attractive than others. Evidence for clear preferences for certain chemotypes by *U. tanaceti* and *M. tanacetaria* comes from laboratory assays, in which unwinged aphids were offered choices between leaves of two chemotypes in different combinations (Jakobs and Müller, 2018; Jakobs and Müller 2019). Aphid choice seems more clear-cut in such restricted conditions than in the field, where several other differences between plant individuals and numerous other environmental factors make the real-time odour environment highly complex, particularly for flying morphs. Nevertheless, higher early colonisation by winged morphs of another aphid species was found in natural conditions for chemotypes emitting α-thujone among other terpenoids (Clancy et al., 2016). Winged morphs of another aphid species infesting Poaceae displayed higher gene expression levels of chemosensory proteins than unwinged ones (Peng et al., 2020), suggesting that high sensitivity towards odour profiles may be expected particularly in these morphs. Second, we had expected a higher attraction of aphids to plants in homogenous plots since these should emit less-mixed odours than heterogenous plots. Once the different plots are approached by the winged individuals, cues deciphering host occupancy, providing information about prior aphid (Mehrparvar et al., 2014) or virus infestations (Eigenbrode et al., 2002), or visual cues (Döring and Chittka, 2007) might become more important for attraction to — or repellence of — certain plant individuals than the volatile terpenoid cues. Such diverse factors may explain, why no preferences for chemotype or plot-type were observed in the present study. Apart from such effects, the distance between plants of different chemical profiles may also be decisive for distinct preference behaviour of winged aphids, as the mixing degree of different plant odours will be affected (Shao et al., 2021). Recently it has been revealed that the exact ratio of compounds in the odour bouquet of a plant is already changing at short distances (Cai et al., 2022), which may affect host plant discrimination, particularly in stands of conspecific neighbourhoods.

No effect on the pure presence but lower counts, i.e. abundances, of winged and unwinged *U. tanaceti* were observed on plants of the Keto and ABThu chemotype in homogenous compared to heterogenous plots, while the opposite pattern was found on plants of the Myrox chemotype. These results suggest that plants of the latter chemotype are more resistant towards *U. tanaceti* than the other tested chemotypes. Also under laboratory conditions, aphids of *U. tanaceti* showed different population growth on plants of different chemotypes (in interaction with plant part) (Jakobs and Müller, 2018), indicating that chemical differences between these plants can be important factors influencing aphid fitness. Apart from host-plant related factors, also top-down effects may have influenced the aphid counts. Indeed, tansy is known to establish chemotype-specific arthropod food webs (Balint et al., 2016). Our results clearly show that not only the individual plant chemotype but also the chemotype composition in the neighbourhood of the respective plant is of importance for the interaction of *T. vulgare* with *U. tanaceti*. The lower abundance of aphids on plants of the Keto and ABThu chemotype in heterogenous compared to homogenous plants supports the associational resistance hypothesis (Tahvanainen and Root, 1972; Randlkofer et al., 2010), which predicts lower herbivore damage in more diverse communities. Diverse neighbourhoods may also harbour more natural enemies, leading to reduced herbivore pressure (Cappuccino et al., 1998). Such associational effects apparently also apply to plant communities of conspecifics that are chemically diverse and may explain why we find high intraspecific chemodiversity in some species. Surprisingly, the associational resistance seems specific for certain chemotypes. For instance, the BThu and Aacet chemotype showed no differences in the abundance of *U. tanaceti* between the plot-types, whereas abundances were even higher on plants growing in heterogenous plots for the Myrox chemotype. Interestingly, plants growing in homo- versus heterogenous plots also showed chemotype-specific differences in relative proportions of certain terpenoids. For example, artemisia alcohol was present in lower proportion in plants of the Myrox chemotype when grown in heterogenous plots, while no difference in the proportion of this terpenoid was found in plants of the Aacet chemotype between different plot types. Chemotype-specific differences in terpenoid composition between plants growing in different plot-types may thus explain the chemotype-specific associational resistance effects on the aphids. Within the chemical information framework it is suggested that specialised plant metabolites act either directly, as repellents or by distracting herbivores (Kessler and Kalske, 2018). Since *U. tanaceti* showed different performance but no different preference in our study system, terpenoids differentially induced in neighbours of specific chemotypes might act as direct chemical defence. Changes in plant defences in dependence of the neighbourhood have been recently also revealed in different tree species (Poeydebat et al., 2021). In particular, terpenoids are known to mediate plant-plant communication by inducing different signalling pathways (Rosenkranz et al., 2021). Moreover, neighbouring tansy plants may benefit in a highly chemotype-specific way from top-down effects, as predators also show different abundances on distinct chemotypes of tansy (Benedek et al., 2019). Furthermore, in our specific set-up all chemotypes may benefit from and/or provide associational resistance towards other antagonists than *U. tanaceti*. Another possible mechanism for associational effects is the enrichment of leaves from neighbouring plants with compounds of lower volatility (Himanen et al., 2010). Whether tansy indeed also adsorbs volatiles from neighbours, which may affect herbivores, remains to be tested.

The three aphid species showed different responsiveness. In contrast to *U. tanaceti*, no effects were observed on the abundance of *M. tancetaria*, but the probability of presence of winged and unwinged aphids was higher on plants of the ABThu chemotype in homogenous compared to heterogenous plots, supporting again associational resistance in this chemotype. When growing in homogenous plots, plants of this chemotype may be particularly supportive for *M. tancetaria.* A higher presence of aphids may lead to an enhanced release of volatiles due to induction, causing even higher colonisation of these plants for aphids, but this idea needs further testing. Impacts of intraspecific differences in groups of metabolites on arthropods and different inducibility have been found for other plant metabolite classes in other plant species. For example, differences in glucosinolate profiles of different populations of *Brassica oleracea* influenced the performance of both herbivores and their parasitoids (Harvey et al., 2011), and those plants showed specific pattern in herbivore-induced concentrations of glucosinolates (Harvey et al., 2011; Gols et al., 2018). Moreover, for aphids, different morphs can also show differences in both physiology and behaviour, even towards the same volatiles (Webster, 2012; Peng et al., 2020). Unwinged morphs may favour a broader range of host cues and are attracted to more volatiles than winged morphs, potentially due to the fact that they do not migrate and thus encounter less non-host cues (Webster, 2012). In our experiment, unwinged aphids may have been most likely offspring from winged aphids that had settled and reproduced but we cannot exclude that also some unwinged aphids moved between plants standing close to each other. In particular, *M. tanacetaria* readily drop from their host plants when being disturbed (personal observation) and may then switch to a close-by neighbour. In contrast to the two other aphid specialists on tansy, the presence and abundance of *M. fuscoviride* was not affected by chemotype or plot-type, but total counts were higher in the presence of ants tending aphid colonies. This finding is in line with previous findings that report positive effects of ant-tendance on the reproduction and other life history traits of *M. fuscoviride* (Flatt and Weisser, 2000). In another study significant effects of tansy chemotypes dominated by either *L*-camphor, (*Z*)-β-terpineol or eucalyptol on the abundance of *M. fuscoviride* were found (Senft et al., 2018). Thus, tritrophic interactions between plants, aphids and ants may be chemotype-specific. Season affected almost all traits of the three aphid species studied here, with peak abundances differing slightly between species. Seasonal variation in plant chemistry as well as continuous changes in induction pattern depending on distinct plant attacks may contribute to this pattern. In addition to this temporal differentiation, the three species also show spatial niche differentiation by colonising different plant parts, with *U. tanaceti* infesting old leaves, *M. tanacetaria* young plant parts (Jakobs et al., 2019) and *M. fuscoviride* prefering stems and inflorescences (Loxdale et al., 2011). Competition has been described between *M. tanacetaria* and *M. fuscoviride*, with the latter being dominated but not entirely suppressed if no ants are present and *vice versa* (Mehrparvar et al., 2018). In our study, a rapid decrease of the count of *M. tanacetaria* was observed followed by a rapid increase of *M. fuscoviride*, indicating possible competition. However, this rapid decrease of *M. tanacetaria* was also observed in chemotype by plot-type combinations in which almost no *M. fuscoviride* were observed. Furthermore, *M. fuscoviride* occupied at maximum half as many plants as *M. tanacetaria*. Thus, competition was likely of no or minor relevance in our study.

Attraction and occurrence of the three aphid species were also analysed with respect to the terpenoid chemodiversity calculated as a gradient rather than distinct chemotype classes. The sampling for this terpenoid profile analysis took place during the peak occurrence of the three aphid species in June and thus data were exclusively related to the aphid traits in that particular week. Chemodiversity, calculated as Shannon index (Hs), had no effects on the presence of winged morphs; however, overall, for *M. tanacetaria* and *M. fuscoviride* only few winged aphids were observed in this week. In contrast, increasing Hs affected the total count of *U. tanaceti* negatively on a plot-level (Hs_plot_), i.e. in relation to β-chemodiversity, but not on an individual level (Hs_ind_), i.e. in relation to α-chemodiversity. It has been proposed that α-chemodiversity depends on the number of interactions an individual plant has, whereas β-chemodiversity might be maintained in order to address e.g. fluctuating adaptations and population sizes by herbivores (Kessler and Kalske, 2018). Increased population level chemodiversity has been found to be associated with a decreased herbivore damage in several studies (Bustos-Segura et al., 2017; Salazar and Marquis, 2022). For example, on *Piper* species particularly negative effects for plots with high levels of high-volatility chemodiversity were found on specialist herbivores (Salazar et al., 2016). The fact that negative effects similar to those observed in interspecifically diverse plots were likewise found for Hs_plot_ of conspecific plots in our tansy system further supports the associational resistance hypothesis in the scope of intraspecific chemical variation.

Interestingly, for *M. tanacetaria* the pattern was different. Here, the total count increased with higher Hs_ind_, while Hs_plot_ showed no significant correlation. The increased Hs_ind_ might in this case not be cause but a consequence of an increased total *M. tanacetaria* count, potentially due to herbivore-induced changes in terpenoids. In fact, an aphid species-specific change in chemical composition has been found in tansy of different chemotypes (Jakobs et al., 2019), but effects on terpenoid profiles by aphid infestation remain to be investigated in this system. Since no correlation was found between *M. tanacetaria* abundance and Hs_plot_, infestation with *M. tanacetaria* may not mediate communication between neighbouring plants to induce chemical defences, as found in other species (Baldwin et al., 2006), or the relationship between the infestation and responses is non-linear. Moreover, we only calculated the chemodiversity of terpenoids, but several other metabolites of other structural classes, particularly of the phloem sap, are highly relevant for determining the performance of aphids (Dreyer and Campbell, 1987; Cao et al., 2018) and may affect the overall structural diversity, next to the compound diversity, of the plants and plots (Whitehead et al., 2021). The mechanisms how chemodiversity in itself provides associational effects might not only be specific to the herbivore morph (winged vs. unwinged) and species, but also to the different metabolites and metabolite classes the host plant species expresses.

In conclusion, we showed that intraspecific chemodiversity in plant communities can provide associational resistance at least against some herbivorous species. To unravel these effects, it is important to analyse each species interaction, considering also developmental stage, with plant chemodiversity in two different forms on two different levels. Firstly, plant chemodiversity can be measured in the form of distinct classes as chemotypes with their specific composition of plant compounds but also in the form of a continuous gradient as a measure of chemodiversity, where different classes of metabolites could be considered. Other indices also consider differences in structural vs. compound diversity (Whitehead et al., 2021) or take into account the biochemical and structural properties of compounds (Petrén et al., 2023). Secondly, chemodiversity can be considered on the level of the individual plant but also summarised on the level of the plant community. In this study, the focus was on the effects of chemodiversity on plant antagonists. Future studies will focus on effects of associational resistance on plant performance by studying growth and fitness traits in these chemically more or less diverse neighbourhoods.

## Data availability statement

The raw and processed field data (aphid scores, morphological characterisation, terpenoid analysis) are stored together with the R scripts in the github repository https://github.com/DoZi93/CommonGarden-aphid-2021 in the folder “/Data”. 

## Author contributions

CM conceived the ideas and designed the common garden set-up. DZ collected and analysed the data and wrote a first draft of the manuscript. CM revised the manuscript. All authors contributed to the article and approved the submitted version.
