# Supplementary material for: Intraspecific chemodiversity provides plant individual- and neighbourhood-mediated associational resistance towards aphids
Source: Front Plant Sci. 2023 Apr 4;14:1145918. doi: 10.3389/fpls.2023.1145918 (PMC10111025; doi:10.3389/fpls.2023.1145918)
Supplement: Supplementary file 1 [file DataSheet_1.pdf]

Supplementary Material for article

Intraspecific Chemodiversity Provides Plant Individual- and Neighbourhood-Mediated Associational Resistance towards Aphids

Dominik Ziaja, Caroline Müller*

*** Correspondence:** Caroline Müller: caroline.mueller@uni-bielefeld.de

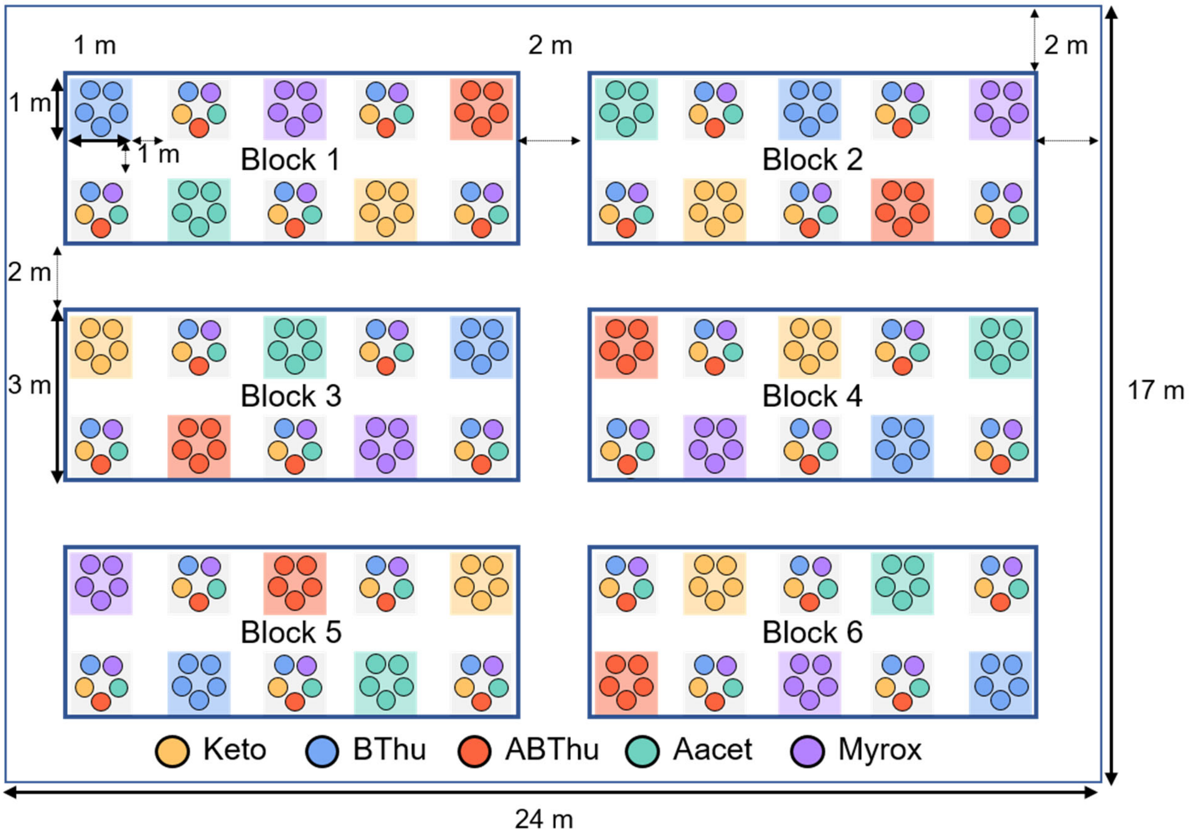

Supplementary Figure 1. Experimental common garden design. Plants of five different chemotypes (Keto: artemisia ketone chemotype, BThu: β-thujone chemotype, ABThu: α-/β-thujone chemotype, Aacet: artemisyl acetate/artemisia ketone/artemisia alcohol chemotype, Myrox: (*Z*)-myroxide/santolina triene/artemisyl acetate chemotype) were planted in plots with all five individuals sharing the same chemotype (homogenous) or all five individuals expressing a different chemotype (heterogenous). The experimental set-up was replicated in six blocks. Number of plants in total = 300. As one plant in a homogenous plot turned out to be not the correct chemotype, only 295 plants (59 plots) were considered for further analysis.

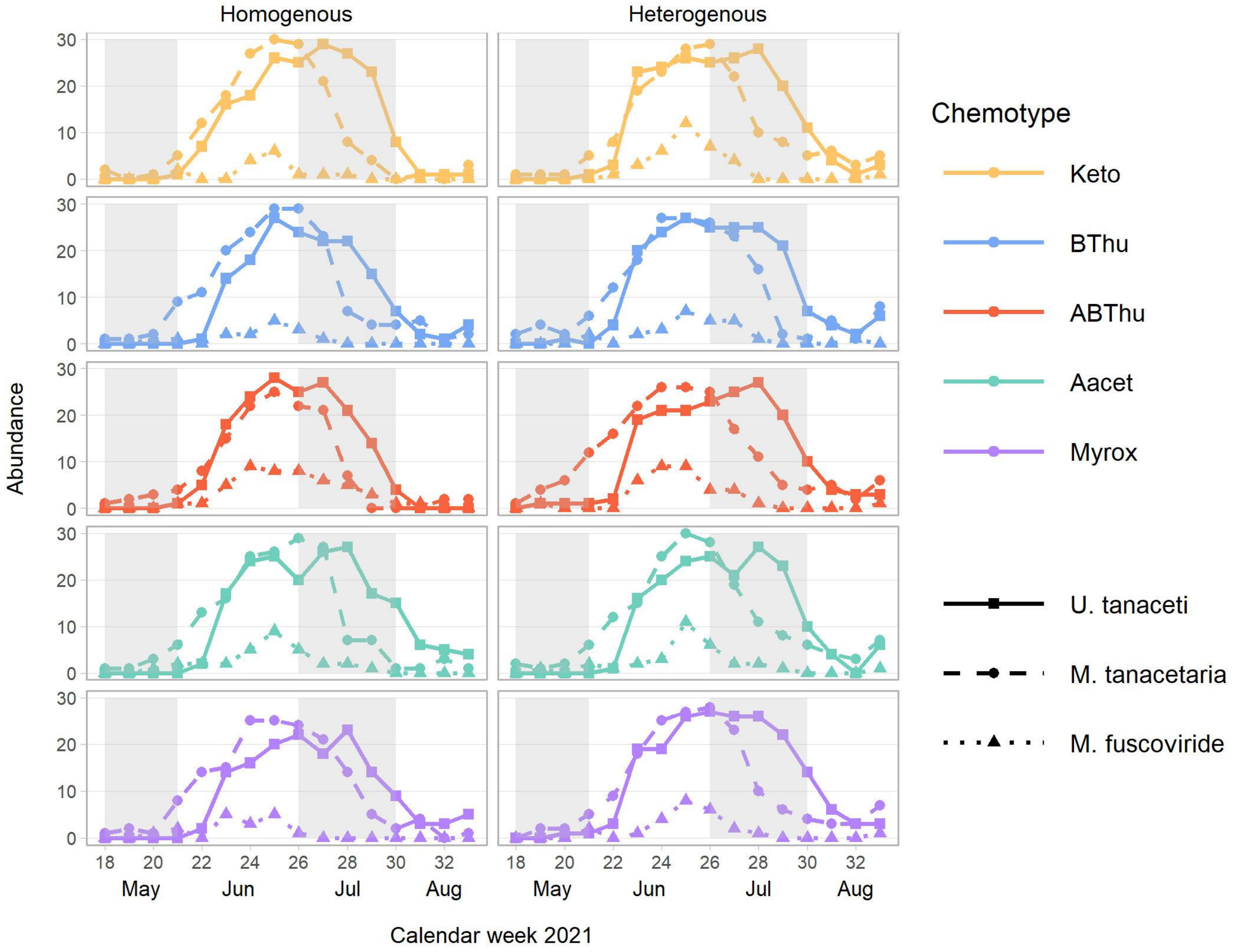

Supplementary Figure 2. Abundance (presence on plant) of winged and unwinged aphids of *Uroleucon tanaceti* (■, solid line), *Macrosiphoniella tanacetaria* (●, dashed line) and *Metopeurum fuscoviride* (▲, dotted line) per calendar week on plants of *Tanacetum vulgare* grown in plots of five individuals of the same (homogenous) or different (heterogenous) chemotypes (Keto: artemisia ketone chemotype, BThu: β-thujone chemotype, ABThu: α-/β-thujone chemotype, Aacet: artemisyl acetate/artemisia ketone/artemisia alcohol chemotype, Myrox: (*Z*)-myroxide/santolina triene/artemisyl acetate chemotype) across the season. n = 25 (plants with Myrox chemotype in homogenous plots) or n = 30 (plants for each of the other combinations of chemotypes and plot-type).

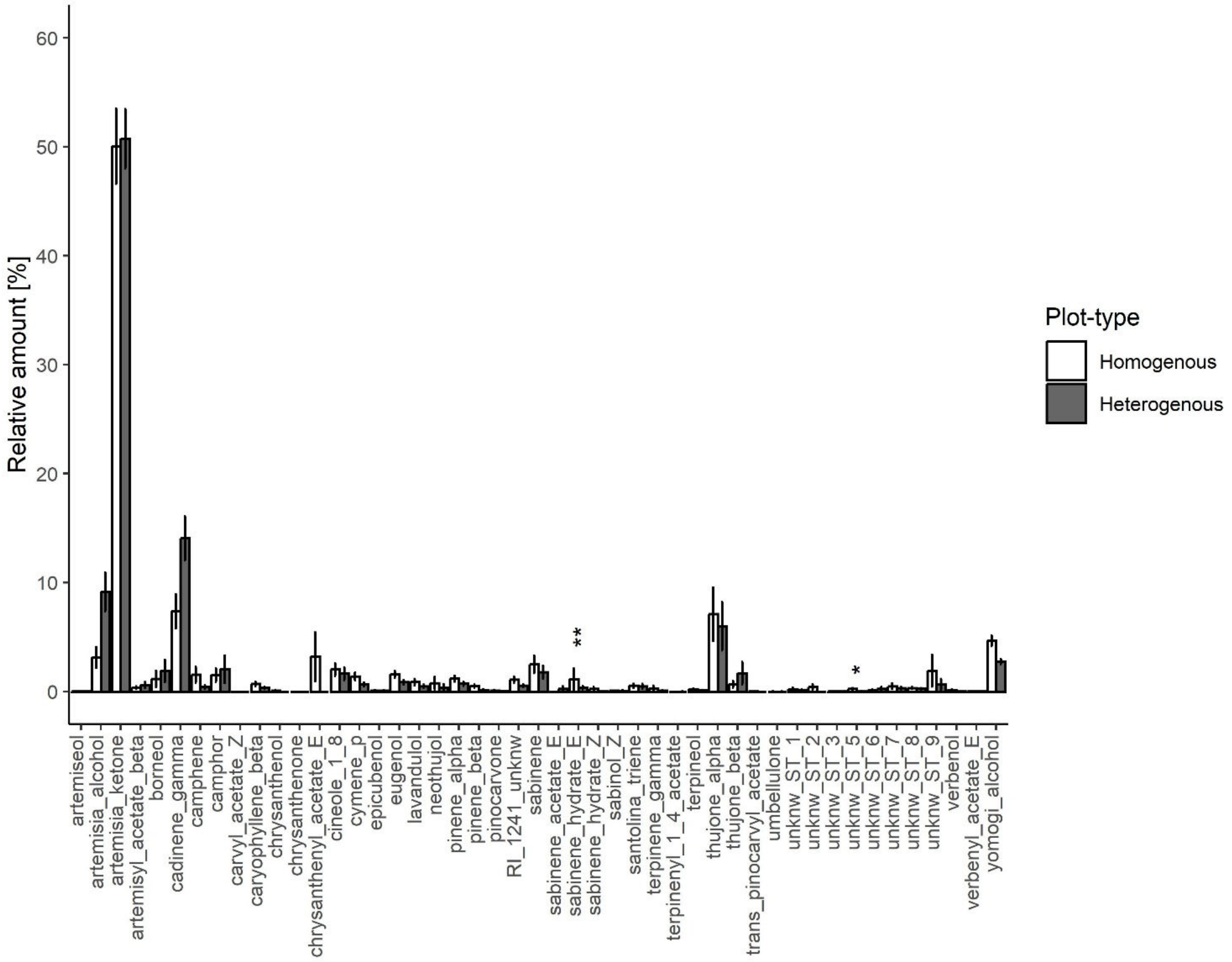

Supplementary Figure 3. Relative proportion of compounds in leaf samples of the artemisia ketone chemotype (Keto) from *Tanacetum vulgare* plants grown in plots consisting of five individuals of the same (homogenous) or different (heterogenous) chemotypes. Leaf samples were collected in June 2021. Shown are the mean ± standard error across n = 30 plants (heterogenous) or n = 29 (homogenous). Significant differences are based on pairwise Mann-Whitney U tests with Holm-method *p*-value correction (*, $p < 0.05$; **, $p < 0.01$). ST = sesquiterpenoid, unknw = unknown, RI = retention index.

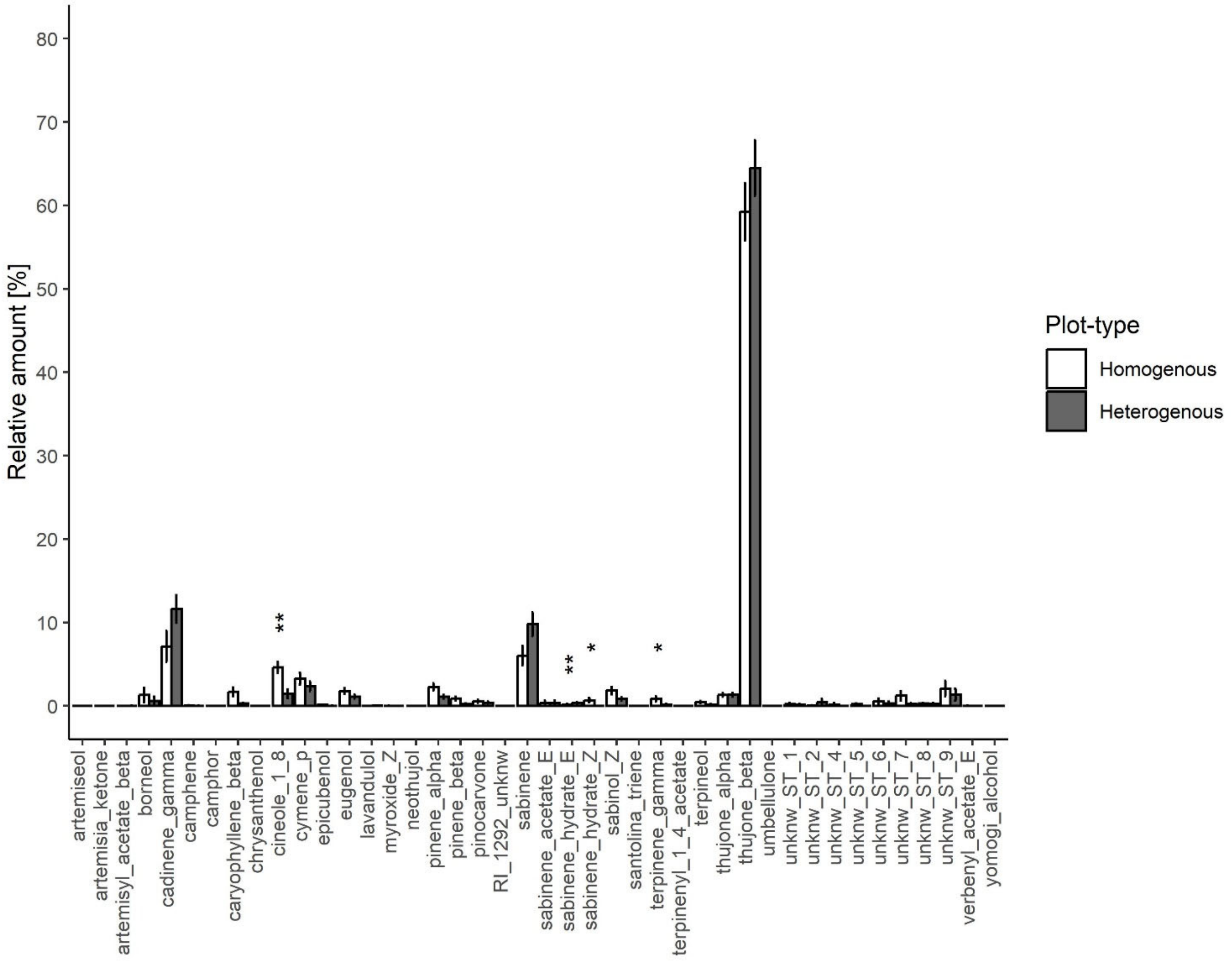

Supplementary Figure 4. Relative proportion of compounds in leaf samples of the β-thujone chemotype (BThu) from *Tanacetum vulgare* plants grown in plots consisting of five individuals of the same (homogenous) or different (heterogenous) chemotypes. Leaf samples were collected in June 2021. Shown are the mean ± standard error across n = 30 plants. Significant differences are based on pairwise Mann-Whitney U tests with Holm-method *p*-value correction (*, $p < 0.05$; **, $p < 0.01$). ST = sesquiterpenoid, unknw = unknown, RI = retention index.

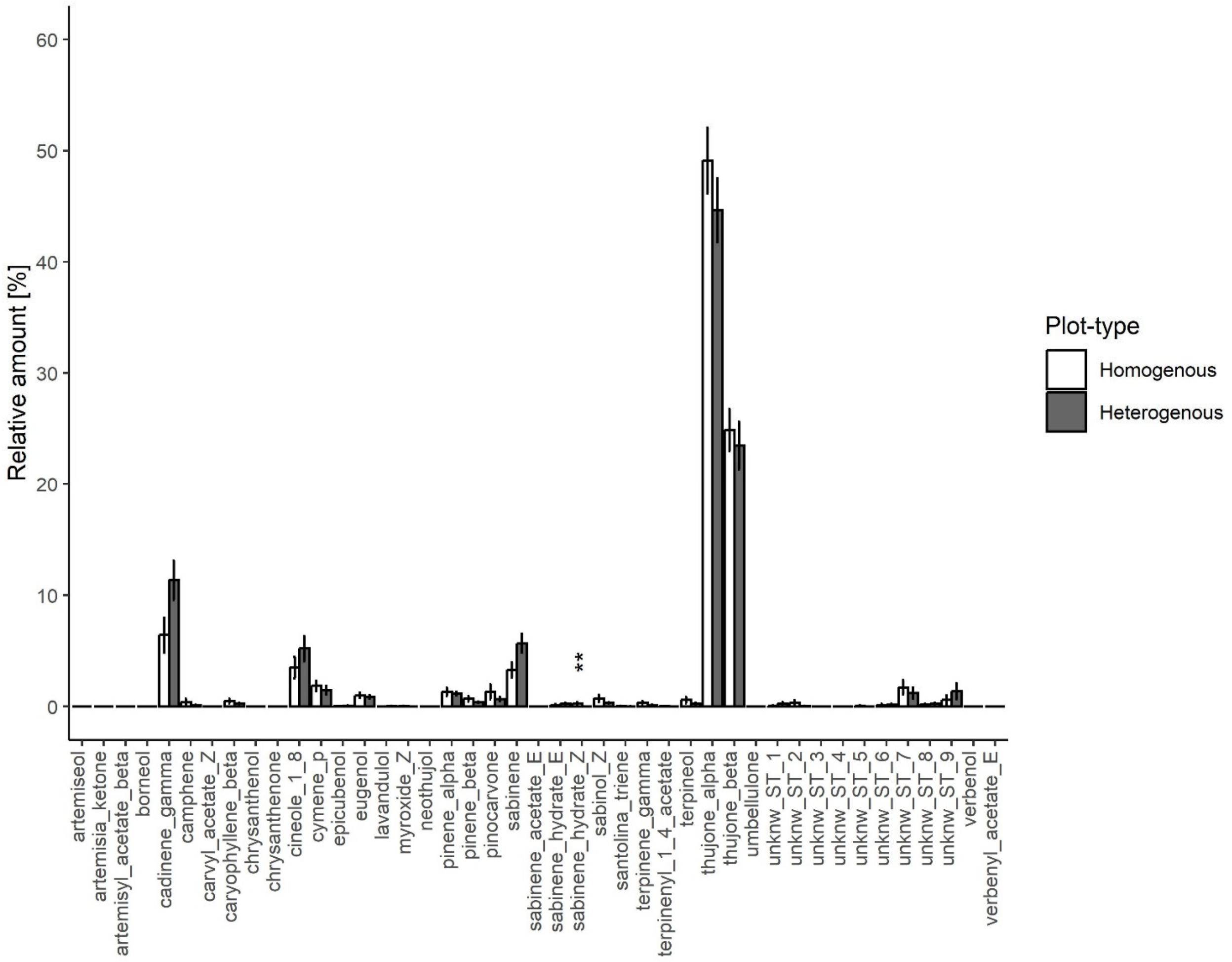

Supplementary Figure 5. Relative proportion of compounds in leaf samples of the α-/β-thujone chemotype (ABThu) from *Tanacetum vulgare* plants grown in plots consisting of five individuals of the same (homogenous) or different (heterogenous) chemotypes. Leaf samples were collected in June 2021. Shown are the mean ± standard error across n = 29 plants. Significant differences are based on pairwise Mann-Whitney U tests with Holm-method *p*-value correction (**, $p < 0.01$). ST = sesquiterpenoid, unknw = unknown, RI = retention index.

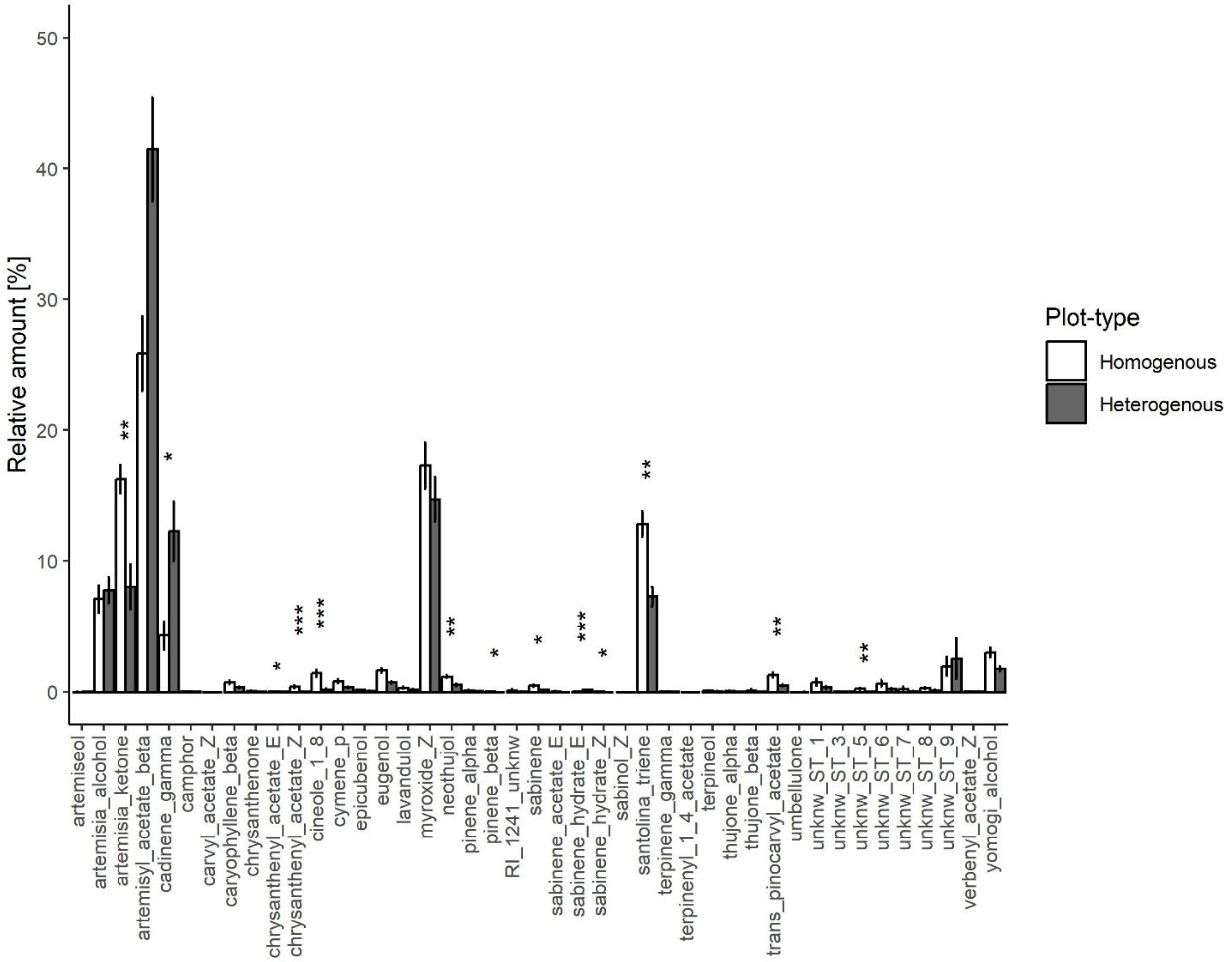

Supplementary Figure 6. Relative proportion of compounds in leaf samples of the artemisyl acetate/artemisia ketone/artemisia alcohol chemotype (Aacet) from *Tanacetum vulgare* plants grown in plots consisting of five individuals of the same (homogenous) or different (heterogenous) chemotypes. Leaf samples were collected in June 2021. Shown are the mean ± standard error across n = 30 plants. Significant differences are based on pairwise Mann-Whitney U tests with Holm-method *p*-value correction (*, $p < 0.05$; **, $p < 0.01$; ***, $p < 0.001$). ST = sesquiterpenoid, unknw = unknown, RI = retention index.

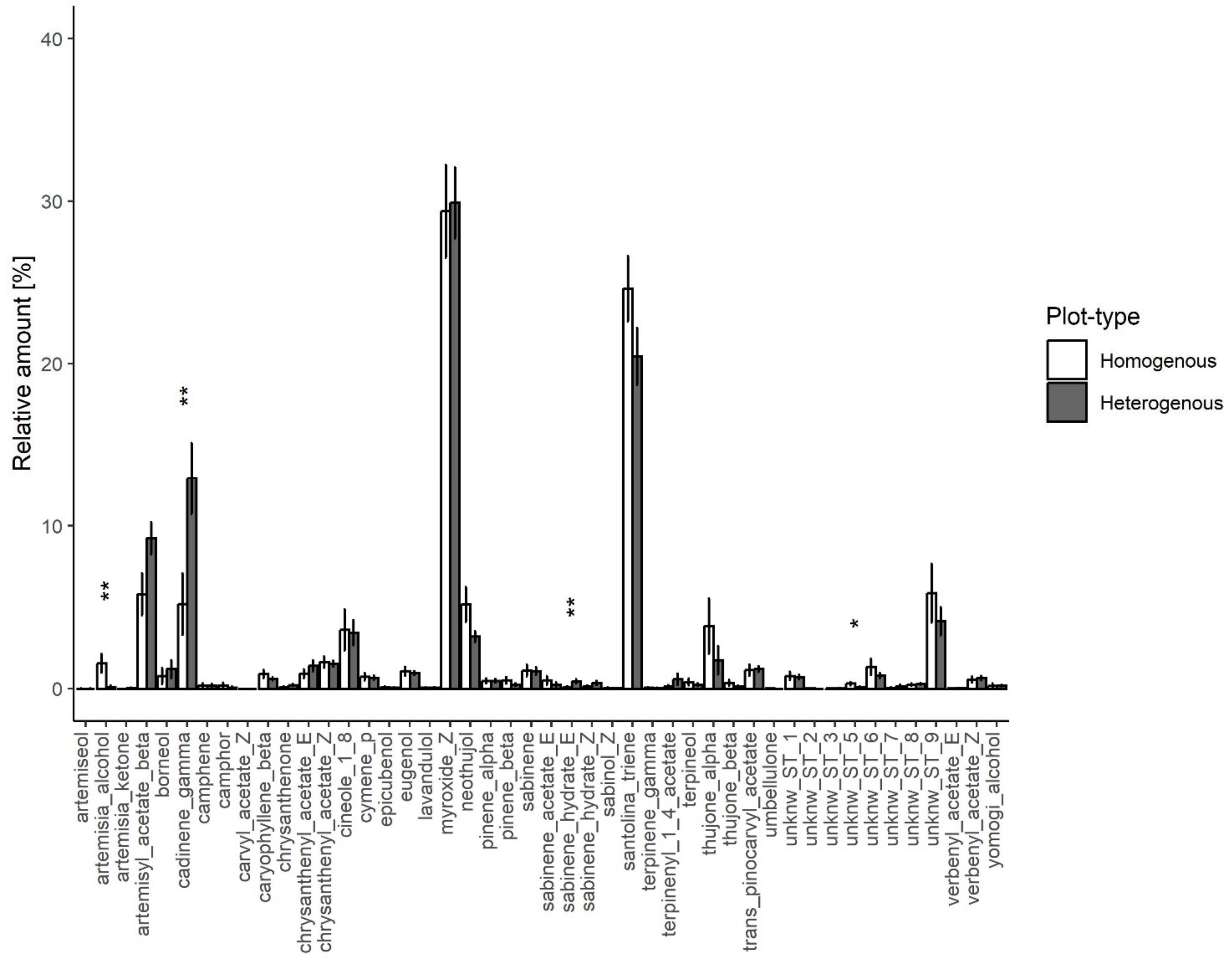

Supplementary Figure 7. Relative proportion of compounds in leaf samples of the (*Z*)-myroxide/santolina triene/artemisyl acetate chemotype (Myrox) from *Tanacetum vulgare* plants grown in plots consisting of five individuals of the same (homogenous) or different (heterogenous) chemotypes. Leaf samples were collected in June 2021. Shown are the mean ± standard error across homogenous (n = 25) or heterogenous (n = 30) plots. Significant differences are based on pairwise Mann-Whitney U tests with Holm-method *p*-value correction (*, $p < 0.05$; **, $p < 0.01$). ST = sesquiterpenoid, unknw = unknown, RI = retention index.
